# Predators of the giant pine scale, *Marchalina hellenica* (Gennadius 1883; Hemiptera: Marchalinidae), out of its natural range in Turkey

**DOI:** 10.1515/biol-2021-0066

**Published:** 2021-07-01

**Authors:** Şükran Oğuzoğlu, Mustafa Avcı, Kahraman İpekdal

**Affiliations:** Department of Forest Engineering/Forest Entomology and Conservation, Isparta University of Applied Sciences, Faculty of Forestry, Isparta, Turkey; Department of Landscape Engineering, Faculty of Agriculture, Ahi Evran University, Kırşehir, Turkey

**Keywords:** honeydew producer, Brutian pine, biological control, natural enemy, prey shift

## Abstract

*Marchalina hellenica* is a sap sucking scale insect endemic to the Aegean basin and it has been introduced to several regions in Greece and Turkey to increase pine honey production. It is also considered as a pest since heavy infestation may leave the host trees vulnerable to secondary pests. An understanding of its natural predators would facilitate planning biocontrol programs. Although there are several studies reporting the predators of *M. hellenica* in its native range, there is no study identifying those in its introduced range. We aimed to determine predators of *M. hellenica* in Burdur, one of its introduced sites in Turkey. We carried out sampling through regular visits in an *M. hellenica*-infested locality nearby Burdur Lake. Through field and laboratory observations, we identified 19 species predating upon *M. hellenica*. Comparing predators reported in previous studies in its native range and those we found in the present study showed that 12 of the species that we found are new reports for the species predating upon *M. hellenica*. The highest number of predator individuals belonged to the monophagous *Neoleucopis kartliana*. *Myrrha octodecimguttata*, *Chilocorus bipustulatus* and *Harmonia quadripunctata* were also the most frequently observed predators.

## 1 Introduction

The giant pine scale, *Marchalina hellenica* (Gennadius 1883; Hemiptera: Marchalinidae), is an economically important species for two contrasting reasons. First, it is the most significant of several honeydew-producing insects in Greece and Turkey; pine honey production relies mainly on *M. hellenica* honeydew in both the countries [1,2]. For this reason, it has been intentionally introduced at many natural pine forests in these countries and its population has increased locally [3,4,5]. Second, it is a pine pest as it feeds on pine sap and can cause increment loss, desiccation, branch dieback, increasing crown transparency and tree decline [5,6,7,8,9]. Furthermore, Petrakis et al. [9] showed that the giant pine scale can cause decrease in diversity of pine-forest-related insects. Therefore, its population size must be in a balance to provide the highest possible honey yield and the lowest possible damage to the pine ecosystem. However, this is not always possible due to climate warming and human-mediated introduction of the species, and it can turn to be a pest which may necessitate control measures to be taken. Being endemic to Greece and Western Turkey, *M. hellenica* has been introduced to the Italian island of Ischia and Australia, where it is accepted as a dangerous exotic species [10,11]. It has also been reported in Armenia, Georgia and Krasnodar and Sochi in Russia [12,13], although taxonomic status of the Caucasian population is controversial [14].

Predators and parasitoids play a significant role in suppressing the population size of forest pest insects [15]. Exotic species usually benefit from the enemy-free advantage of the new ecosystems during the early stages of the invasion [16]. This advantage can subside as the host-specific parasitoids and predators follow the invader or the generalist parasitoids and predators start to feed on the invader. Generalist parasitoids and predators can use “host/prey switching” strategy; thus, they can forage on the most abundant prey, the invader in the case of an invasion, in a habitat (e.g., ref. [17]).

**Figure A1:**
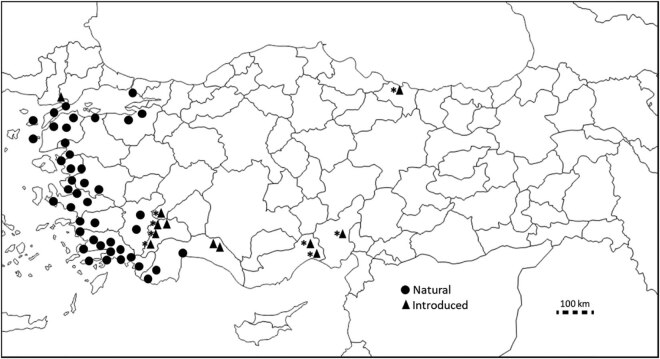
Natural (circles) and introduced (triangles) distribution of *Marchalina hellenica* in Turkey (data from ref. [ 4,18,19,24] and personal observations) along with the first records made in the present study (asterisks) (counter map from ref. [87]).

The giant pine scale occurs almost in the entire western coasts of Turkey [4,18,19]. It has also been introduced into several regions in the country by beekeepers (Figure A1). Burdur basin, where the present study was undertaken, is one of those regions. Although the first report of *M. hellenica* in the region was in 2004 [20], the local beekeepers introduced the giant pine scale-infested branches into the region in the early 1990s from Muğla province ca. 200 km west of the study site (M. Bilgiç/Burdur Association of Beekeepers, personal communication). The species has been well established in this isolated basin during the last 30 years. However, it has not been investigated which predatory species prey upon *M. hellenica* during this period in its recent range. This information may be important for biological control against *M. hellenica*. Studies on the predators of the giant pine scale revealed 28 predaceous species (two of which being ectoparasitic) so far in Georgia, Greece and Turkey ([13,14,19] Süreyya and Hovasse 1931 cited in ref. [21,22,23,24]) with 3, 7 and 22 species, respectively (Table A1). Although the striking difference in the number of described predatory species among the three countries could be related to the number of studies conducted in each country, the natural history of *M. hellenica* or a combination of these factors, it is not in our scope to resolve this issue. However, our preliminary observations suggested that the list of predacious species from Turkey seems to be even longer. Accordingly, we asked the following questions in the present study: (1) What are the arthropod predator species in Burdur basin where *M. hellenica* has been an introduced species? (2) How different is the predator composition in the introduced range of *M. hellenica* from those in its natural range?

## 2 Materials and methods

**Figure 1:**
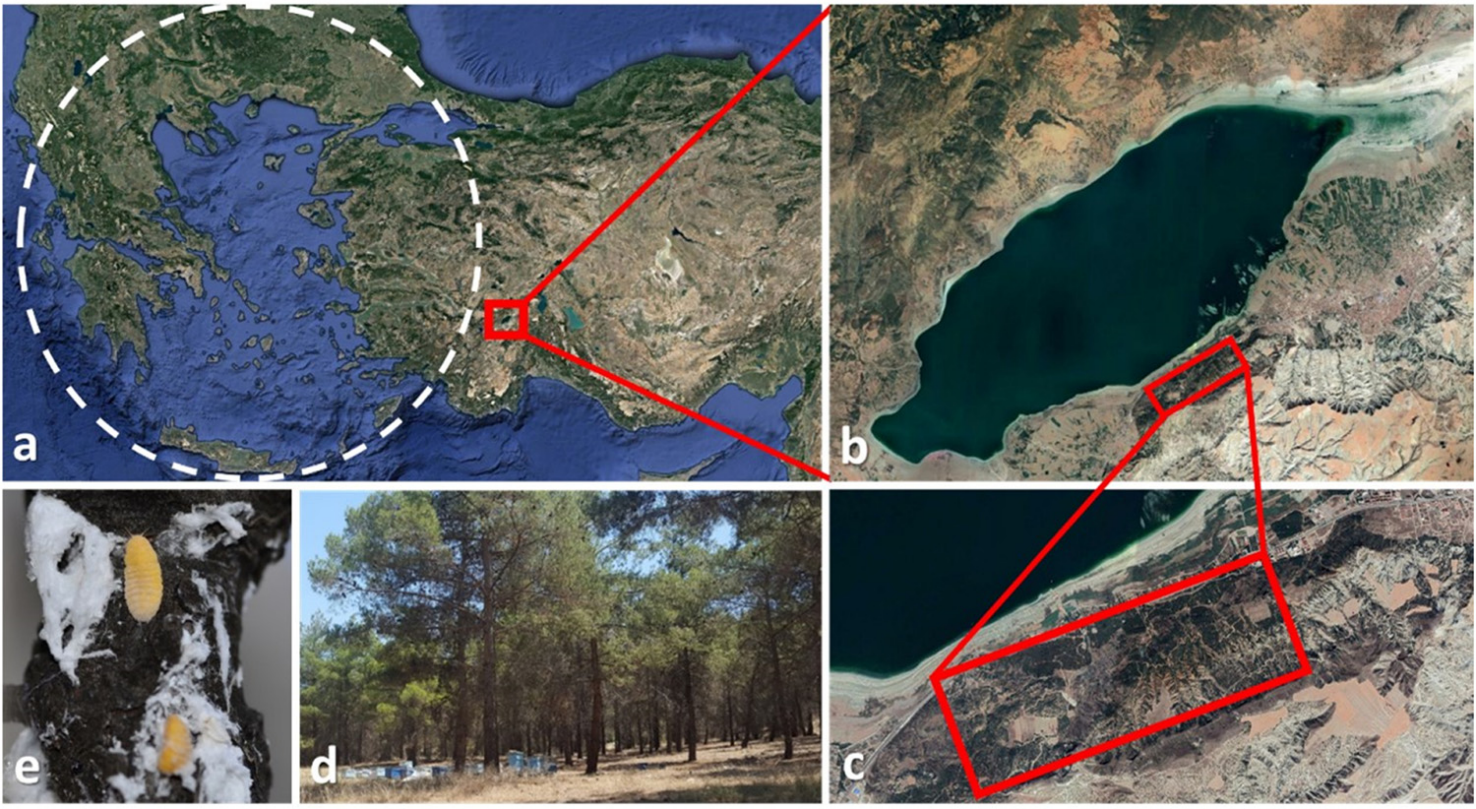
Study site. Dashed circle depicts the approximate natural distribution of *Marchalina hellenica* (a), Burdur Lake (b) and the study site near the lake (c), beehives in *Pinus brutia* stand in the study site (d) and *M. hellenica* adults on the stem bark (e).

The study area, Burdur Urban Forest (Southwestern Turkey), is located at an average altitude of 910 m (a.s.l., ±30 m; 37°41′30″N, 30°11′54″E) and at the northwestern aspect facing the lake (Figure 1). It is an isolated area of ca. 100 ha, consisting of mainly *Pinus brutia*, but also of *P. nigra* and *P. pinea*. Approximately 300 beehives have been placed in the area for pine honey production. Giant pine scale introductions were carried out intensely in the early 1990s in the region. The Brutian pine in the area was heavily infested not only by *M. hellenica* but also by bark beetles (mainly Mediterranean pine beetle, *Orthotomicus erosus* and pine shoot beetle, *Tomicus destruens*).

We identified one site of heavy infestation around Burdur Lake in Burdur basin through visual inspection and visited this site (ca. 5 ha, 37°42′0.81″N–30°12′52.32″E; 37°41′49.78″N–30°12′27.65″E; 37°41′36.83″N–30°11′52.99″E; 37°41′26.94″N–30°11′44.08″E; 37°41′26.22″N–30°11′29.60″E) once in October 2017, 13 times between April and October 2018 and 5 times between March and May 2019 (a total of 19 visits). We always conducted the field visits between 11:00 am and 02:00 pm and during this period, we visited 30 trees which we randomly selected in each visit. We collected adult insects from the infested branches by using an entomological beating sheet and a mouth aspirator. We took advantage of the trap-logs placed in the stand in 2019 by the local department of forestry as a part of the management practices applied against *O. erosus.* We recorded and collected the insects preying on *M. hellenica* on the trap-logs. During the field observations, we also found pupae on the cottony secretions of *M. hellenica*. In order to identify them, we reared these pupae in plastic cages (680 mm × 465 mm × 360 mm, 80 lt) with a ventilated lid in the laboratory and recorded the adult emergences. Then we offered *M. hellenica* nymphs and adults to the adults emerging from the pupae collected from the field to observe predation in the laboratory. Although this was not a setting designed for a choice experiment, it provided us a chance to evaluate possible predation by the species whose immature stages were found in *M. hellenica*-related structures. Finally, we evaluated the collected species according to the relevant literature, and we discarded those which have not been shown to feed on Coccoidea. Thus, we provided evidence for predation through direct observation in the field and the laboratory, and literature records.

The criteria we used to determine predation on *M. hellenica* were as follows: (1) If the potential predator was seen feeding directly on *M. hellenica* (but not on its honeydew) in the field, then we identified the species as a predator. If not, we did not collect it. (2) If it was seen feeding directly on *M. hellenica* in the laboratory, then we identified it as a predator. If not, we discarded it. (3) If the predation could not be detected through field and laboratory observations but was previously reported in the literature, then we identified the considered species as a predator.

Taxonomical identification of the specimens confirmed by specialists is acknowledged at the end of the article. Specimens were stored at the Entomological Museum of Isparta University of Applied Sciences, Faculty of Forestry.

## 3 Results

**Figure 2:**
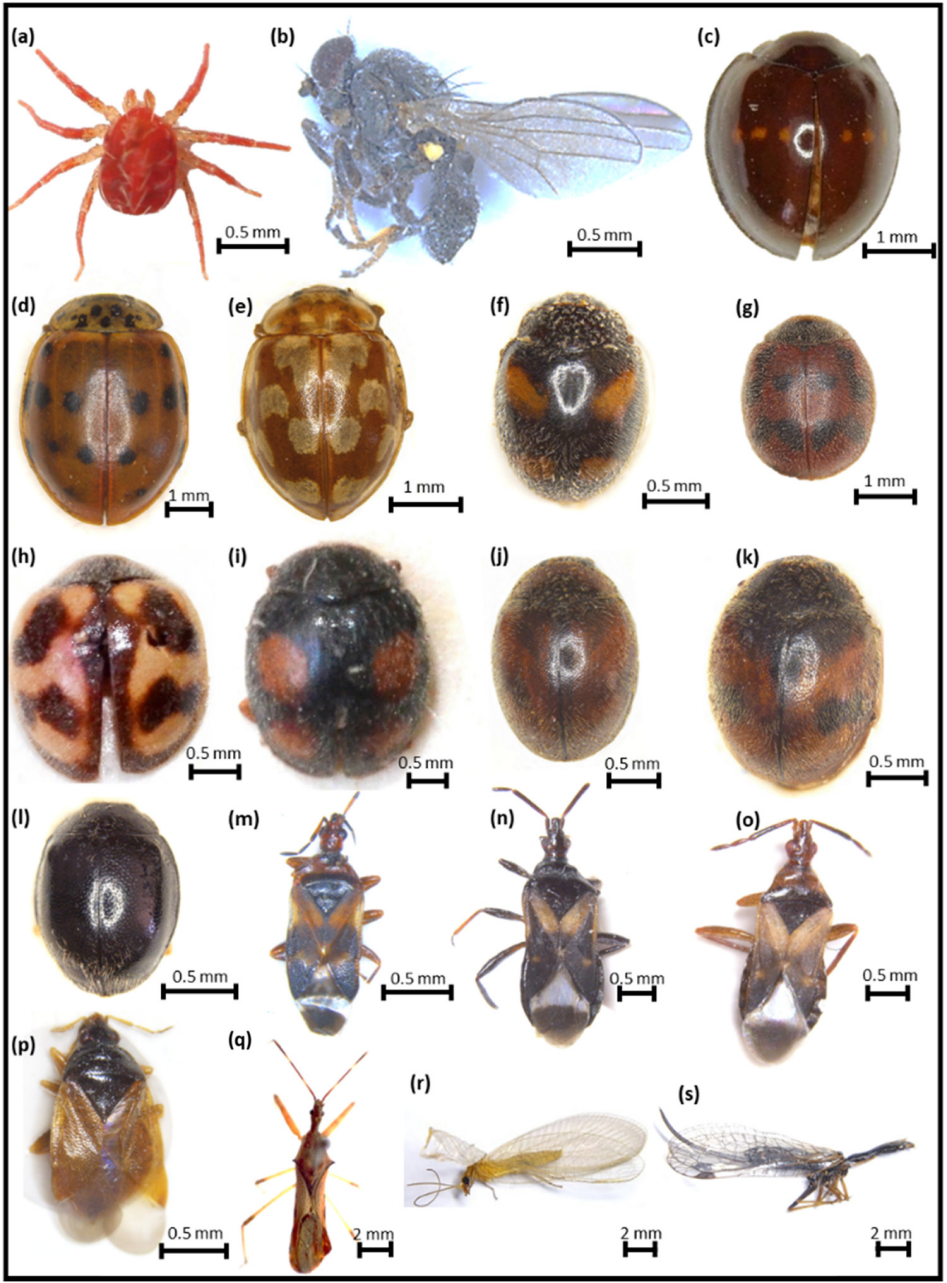
Predators of *Marchalina hellenica* identified in Burdur, Turkey: (a) *Anystis baccarum*, (b) *Neoleucopis kartliana*, (c) *Chilocorus bipustulatus* [88], (d) *Harmonia quadripunctata*, (e) *Myrrha octodecimguttata*, (f) *Nephus quadrimaculatus*, (g) *Novius cruentatus*, (h) *Rodolia cardinalis*, (i) *Scymnus pallipediformis*, (j) *S. subvillosus*, (k) *S. syriacus*, (l) *Stethorus gilvifrons*, (m) *Anthocoris nemoralis*, (n) *A. nemorum*, (o) *Temnostethus reduvinus*, (p) *Orius majusculus*, (q) *Nagusta goedelii*, (r) *Chrysoperla carnea* and (s) *Raphidia ambigua.*

**Figure 3:**
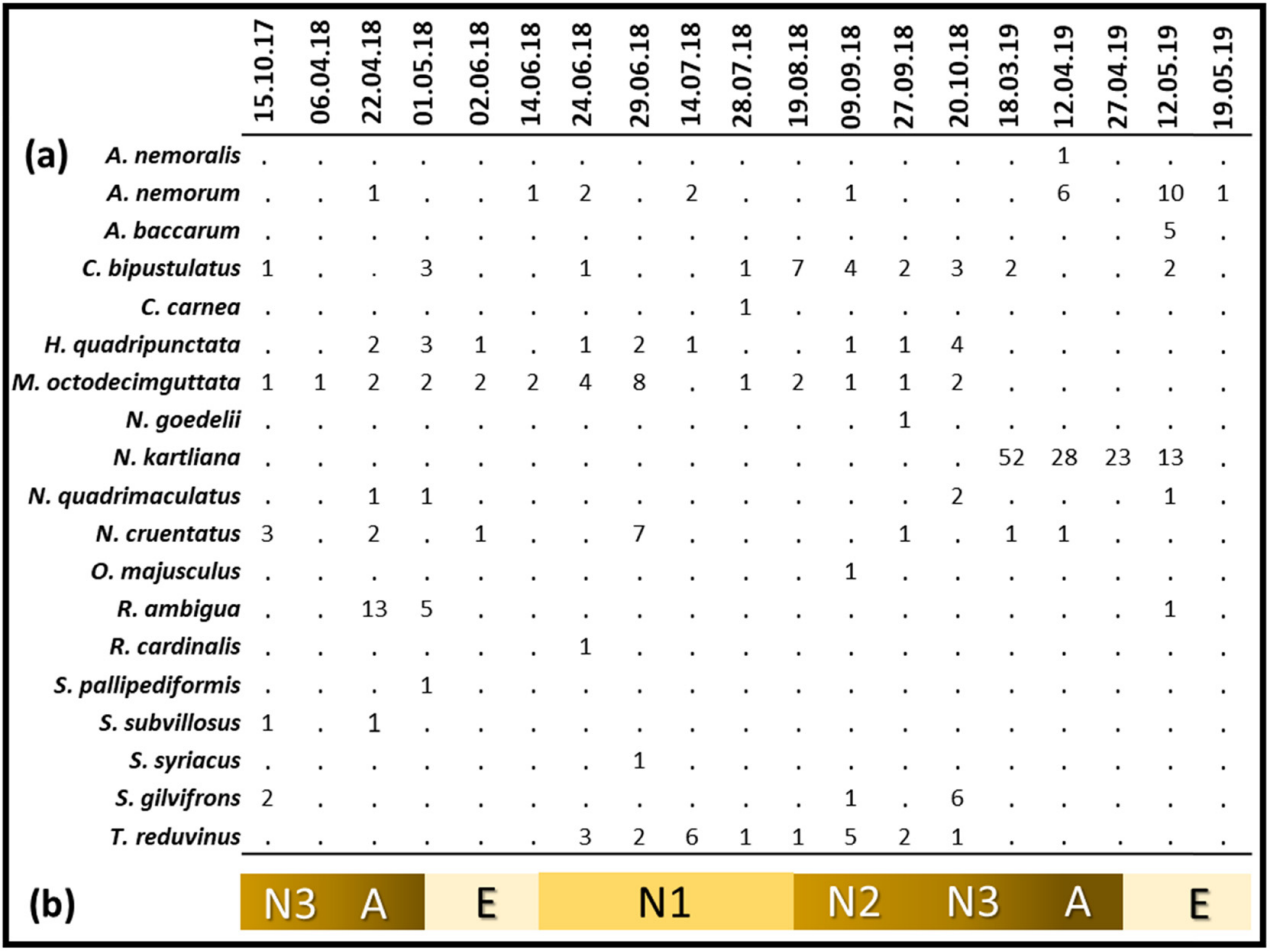
(a) Number of predator individuals per species per field visit. (b) Seasonal development of *M. hellenica* in the region (A: Adult, E: Egg and N: Nymph).

We found 29 insect species during the study, but we eliminated ten species that do not normally prey on scale insects including the giant pine scale. As a result of field and laboratory observations, we identified 19 species (295 individuals) as predators of *M. hellenica*. Among them, one belonged to Acari, ten to Coleoptera, one to Diptera, five to Hemiptera and two to Neuroptera (Table 1, Figure 2). Detailed descriptions of the identified species are given below. Among all the predator species, the one with the highest number of individuals, both in the field and laboratory observations, was *Neoleucopis kartliana* (116 individuals), which was followed by *Myrrha octodecimguttata* (29), *Chilocorus bipustulatus* (26), *Anthocoris nemorum* (24) and *Temnostethus reduvinus* (21) (Table 1). The most frequently detected species was *M. octodecimguttata* which was recorded as 68.4% of all field visits. Other frequently detected species were *C. bipustulatus* (57.9%), *Harmonia quadripunctata* (47.4%), *A. nemorum* (42.1%) and *T. reduvinus* (42.1%) (Table 1). The highest number of predator species (7) was recorded on 22 April 2018 and 9 September 2018; whereas the highest number of predator individuals (55) was recorded on 18 March 2019, which was followed by the record on 12 April 2019 (36) and 12 May 2019 (32) (Figure 3). The lowest number of predator species (1) was recorded on 6 April 2018, 27 April 2019 and 19 May 2019; whereas the lowest number of predator individuals (1) was recorded on 6 April 2018 and 19 May 2019 (Figure 3).

**Table 1:** Predators of *Marchalina hellenica* found in Burdur, Turkey

No	Order	Family	Species	Number of individuals	Individual detection rate (%)^*^	Species detection rate (%)^**^
1	Acari	Anystidae	*Anystis baccarum* ^‡^	5	1.7	5.3
2	Diptera	Chamaemyiidae	*Neoleucopis kartliana*	116	39.3	21.1
3	Coleoptera	Coccinellidae	*Chilocorus bipustulatus* ^‡^	26	8.8	57.9
4			*Harmonia quadripunctata*	16	5.4	47.4
5			*Myrrha octodecimguttata* ^‡^	29	9.8	68.4
6			*Nephus quadrimaculatus* ^+‡^	5	1.7	21.1
7			*Novius cruentatus* ^+‡^	16	5.4	36.8
8			*Rodolia cardinalis* ^‡^	1	0.3	5.3
9			*Scymnus pallipediformis* ^+‡^	1	0.3	5.3
10			*Scymnus subvillosus*	2	0.7	5.3
11			*Scymnus syriacus* ^+‡^	1	0.3	5.3
12			*Stethorus gilvifrons* ^+‡^	9	3.1	15.8
13	Hemiptera	Anthocoridae	*Anthocoris nemoralis* ^+‡^	1	0.3	5.3
14			*Anthocoris nemorum* ^+‡^	24	8.1	42.1
15			*Orius majusculus* ^+‡^	1	0.3	5.3
16			*Temnostethus reduvinus* ^+‡^	21	7.1	42.1
17		Reduviidae	*Nagusta goedelii* ^+‡^	1	0.3	5.3
18	Neuroptera	Chrysopidae	*Chrysoperla carnea* ^+‡^	1	0.3	5.3
19	Raphidioptera	Raphidiidae	*Raphidia ambigua* ^+^	19	6.4	5.3

^*^Percentage of detection of the individuals among all other species.

^**^Percentage of detection of the species in all field visits.

^+^New record as a predator of *M. hellenica.*

^‡^New record for Burdur region.

ACARI: ANYSTIDAE

***Anystis baccarum***(Linnaeus 1758)

Material examined: 12.05.2019 (5 individuals)

Polyphagous predator is known to feed on *M. hellenica* [19]. In Turkey, it occurs in Denizli and Manisa [25], İzmir [26] and Muğla [19]. This is the first report from Burdur.

COLOPTERA: COCCINELLIDAE

***Chilocorus bipustulatus*** (Linnaeus 1758)

Material examined: 15.10.2017 (1), 01.05.2018 (3), 24.06.2018 (1), 28.07.2018 (1), 19.08.2018 (7), 09.09.2018 (4), 27.09.2018 (2), 20.10.2018 (3), 18.03,2019 (2) and 12.05.2019 (2) (26 individuals)

It is known to feed on Coccoidea species [27,28]. In Turkey, it occurs in Aydın, Artvin Denizli, İzmir, Muğla [28], Kahramanmaraş [29], Ankara [30], Antalya [31,32] and Çanakkale [33]. This is the first report from Burdur.

***Harmonia quadripunctata*** (Pontoppidan 1763)

Material examined: 22.04.2018 (2), 01.05.2018 (3), 02.06.2018 (1), 24.06.2018 (1), 29.06.2018 (2), 14.07.2018 (1), 09.09.2018 (1), 27.09.2018 (1) and 20.10.2018 (4) (16 individuals)

It is known to feed on *Torosaspis cedricola* (Balachowsky & Alkan) [34] and *M. hellenica* [24]. In Turkey, it occurs in Adana, Ankara, Afyonkarahisar, Bursa, Denizli, Isparta, [28], Kahramanmaraş [29], Artvin [35], Antalya [31,32], Çanakkale [33], Bartın [36] and Burdur [37].

***Myrrha***(***Myrrha***)***octodecimguttata*** (Linnaeus 1758)

Material examined: 15.10.2017 (1), 06.04.2018 (1), 22.04.2018 (2), 01.05.2018 (2), 02.06.2018 (2), 14.06.2018 (2), 24.06.2018 (4), 29.06.2018 (8), 28.07.2018 (1), 19.08.2018 (2), 09.09.2018 (1), 27.09.2018 (1) and 20.10.2018 (2) (29 individuals)

It is known to feed on *M. hellenica* [19], *Matsucoccus pini* (Green) (Hemiptera: Matsucoccidae) [38], *Carulaspis juniperi* (Bouché 1851) and *Leucaspis lowi* Colvée 1882 (Hemiptera: Diaspididae) [39]. In Turkey, it occurs in Adana, Ankara, Afyonkarahisar, Bursa, Denizli, Isparta, Rize, [28], Kahramanmaraş [29], Balıkesir, Çanakkale, İzmir and Muğla [19]. This is the first report from Burdur.

***Nephus (Nephus) quadrimaculatus*** (Herbst 1783)

Material examined: 22.04.2018 (1), 01.05.2018 (1), 20.10.2018 (2) and 12.05.2019 (1) (5 individuals)

It is known to feed on *Palaeococcus fuscipennis* (Hemiptera: Monophlebidae) (Burmeister 1835) [40] and *Coccus pseudomagnoliarum* (Kuwana 1914) [41,47,84]. This is the first report showing that it predates upon *M. hellenica*. In Turkey, it occurs in Antalya [32] and Diyarbakır [42]. This is the first report from Burdur.

***Novius cruentatus*** (Mulsant 1850)

Material examined: 15.10.2017 (3), 22.04.2018 (2), 02.06.2018 (1), 29.06.2018 (7), 27.09.2018 (1), 18.03.2019 (1) and 12.04.2019 (1) (16 individuals)

It is known to feed on *Palaeococcus fuscipennis* [43]. This is the first report showing that it predates upon *M. hellenica*. Löbl and Smetana [83] reported the species in Turkey without any locality detail. This is the first report from Burdur.

***Rodolia cardinalis***(Mulsant 1850)

Material examined: 24.06.2018 (1 individual)

It is known to feed on *M. hellenica* [19,78] and *P. fuscipennis* [40,43]. In Turkey, it occurs in Mediterranean region [28], Aydın, İzmir, Muğla [19] and Antalya [32]. This is the first report from Burdur.

***Scymnus pallipediformis***Günther 1958

Material examined: 01.05.2018 (1 individual)

It is a polyphagous species feeding on aphids and scale insects [29,42,51]. This is the first report showing that it predates upon *M. hellenica*. In Turkey, it occurs in Mediterranean region [28], Adıyaman, Diyarbakır, Şanlıurfa [42], Ağrı [44], Antalya [31,32], Çanakkale [33], Kahramanmaraş [29], İzmir [45] and Yalova [46]. This is the first report from Burdur.

***Scymnus***(***Pullus***)***subvillosus***(Goeze 1777)

Material examined: 15.10.2018 (1) and 22.04.2018 (1) (2 individuals)

It is a polyphagous species feeding on aphids and scale insects [29,42,51]. It is known to feed on *M. hellenica* [19], *Coccus pseudomagnoliarum* [47] and *Planococcus citri* (Risso) (Hemiptera: Pseudococcidae) [48]. In Turkey, it occurs in Adıyaman, Diyarbakır [42], Antalya [32], Aydın, Balıkesir, İzmir, Muğla [19], Burdur [49], Çanakkale [33], Kahramanmaraş [29] and Yalova [46].

***Scymnus syriacus***(Marsuel 1868)

Material examined: 29.06.2018 (1 individual)

It is known to feed on Aphididae [42,50], Cicadellidae, Coccidae, Diaspididae and Psyllidae [51,52]. This is the first report showing that it predates upon *M. hellenica*. In Turkey, it occurs in Adana, Hatay, Mersin [28], Adıyaman, Şanlıurfa [51], Antalya [32], Diyarbakır [42] and Kahramanmaraş [29]. This is the first report from Burdur.

***Stethorus gilvifrons***(Mulsant 1850)

Material examined: 15.10.2017 (2), 09.09.2018 (1) and 20.10.2018 (6) (9 individuals)

It is known to feed on aphids and scale insects [29,31,51] and Tetranychidae [28]. *Anapulvinaria pistaciae* (Bodenheimer 1926), *Eulecanium rugulosum* (Arch. 1937; Coccidae), *Pistaciaspis pistaciae* Arch. and *Suturaspis pistaciae* (Lindinger 1906) (Diaspididae) are reported to be its prey species [51]. This is the first report showing that it predates upon *M. hellenica*. In Turkey, it occurs in Mediterranean region and Adıyaman, Diyarbakır, Şanlıurfa [42], Antalya [31,32], Çanakkale [33], Isparta [53], İzmir [28] and Kahramanmaraş [29]. This is the first report from Burdur.

DIPTERA: CHAMAEMYIIDAE

***Neoleucopis kartliana*** (Tanasijtshuk 1986)

Material examined: 18.03.2019 (52), 12.04.2019 (28), 27.04.2019 (23) and 12.05.2019 (13) (116 individuals). It feeds only on *Marchalina* [19,54]. In Turkey, it occurs in Antalya, Aydın, Balıkesir, Burdur, Bursa, Çanakkale, Denizli, Edirne, İstanbul, İzmir, Manisa and Muğla [19].

HEMIPTERA: ANTHOCORIDAE

***Anthocoris nemoralis*** (Fabricius 1794)

Material examined: 12.04.2019 (1 individual)

It is a polyphagous species feeding on aphids, mites, psyllids and lepidopteran eggs and young larva [55,56,57]. This is the first report showing that it predates upon *M. hellenica*. In Turkey, it occurs in Antalya, Erzincan, Erzurum, İzmir, Manisa and Mersin [58,59]. This is the first report from Burdur.

***Anthocoris nemorum*** (Linnaeus 1761)

Material examined: 22.04.2017 (1), 14.06.2018 (1), 24.06.2018 (2), 14.07.2018 (2), 09.09.2018 (1), 12.04.2019 (6), 12.05.2019 (10) and 19.05.2019 (1) (24 individuals)

It is a polyphagous species reported to feed on *Cacopsylla pyri* (L.) (Hemiptera: Psyllidae) and *Brevicoryne brassicae* (L.) (Hemiptera: Aphididae) [56,85,86]. This is the first report showing that it predates upon *M. hellenica*. In Turkey, it occurs in Antalya and Erzurum [59], Artvin, Bolu and Trabzon [60]. This is the first report from Burdur.

***Orius***(***Heterorius***)***majusculus*** (Reuter 1879)

Material examined: 09.09.2018 (1 individual)

It is a polyphagous species [61] reported to feed on aphids, thrips and whiteflies [62,63,64]. This is the first report showing that it predates upon *M. hellenica*. In Turkey, it occurs in Eastern Black Sea and Eastern and Central Anatolia regions [60]. This is the first report from Burdur.

***Temnostethus***(***Ectemnus***)***reduvinus*** (Herrich-Schäffer 1850)

Material examined: 24.06.2018 (3), 29.06.2018 (2), 14.07.2018 (6), 28.07.2018 (1), 19.08.2018 (1), 09.09.2018 (5), 27.09.2018 (2) and 20.10.2018 (1) (21 individuals)

It is known to feed on *Lepidosaphes ulmi* (L.) (Diaspididae) and *Agonoscena pistaciae* Burck. & Laut. (Psyllidae) [65,66,67]. This is the first report showing that it predates upon *M. hellenica*. In Turkey, it occurs in Adıyaman, Siirt [67], Antalya, Artvin, Erzincan, Erzurum and Kars [59]. This is the first report from Burdur.

HEMIPTERA: REDUVIIDAE

***Nagusta goedelii*** (Kolenati 1857)

Material examined: 27.09.2018 (1 individual)

It is a polyphagous species reported to feed on aphids, psyllids and pseudococcids [32,68,69,70]. This is the first report showing that it predates upon *M. hellenica*. In Turkey, it has a wide distribution [71,72,73]. This is the first report from Burdur.

NEUROPTERA: CHRYSOPIDAE

***Chrysoperla carnea*** (Stephens 1836)

Material examined: 28.07.2018 (1 individual)

It is a polyphagous species reported to feed on aphids, scale insects, lepidopter eggs and larvae, psyllids, chrysomelid larvae, thrips and acars [74]. This is the first report showing that it predates upon *M. hellenica*. Ülgentürk et al. [19] reported another species of the genus (*C. lucasina*) as a predator of *M. hellenica* in Turkey. It occurs widely in Turkey [75,76]. This is the first report from Burdur.

RAPHIDIOPTERA: RAPHIDIIDAE

***Raphidia ambigua*** Aspöck&Aspöck 1964

Material examined: 22.04.2018 (13), 01.05.2018 (5) and 12.05.2019 (1) (19 individuals)

It feeds on Coccoidea and Raphidiidae [77]. This is the first report showing that it predates upon *M. hellenica*. Argyriou et al. [78] reported another species of the genus (*R. notata* F.) as a predator of *M. hellenica* in Greece. In Turkey, it occurs in Afyonkarahisar, Ankara, Antalya, Balıkesir, Bilecik, Bitlis, Burdur, Çorum, Denizli, Diyarbakır, Elazığ, Isparta, İzmir, Kırşehir, Konya, Mersin, Muğla and Muş [79].

## 4 Discussion

In the present study, we identified 19 species foraging on *M. hellenica* in an isolated *P. brutia* stand, where *M. hellenica* is an introduced species. We report 12 of these species as the predators of *M. hellenica* for the first time (*A. nemoralis*, *A. nemorum*, *C. carnea*, *N. quadrimaculatus*, *N. cruentatus*, *N. goedelii*, *O. majusculus*, *R. ambigua*, *S. pallipediformis*, *S. syriacus*, *S. gilvifrons* and *T. reduvinus*), whereas 12 of the identified species were the first records for the Burdur region (*A. nemoralis*, *A. nemorum, A. baccarum, C. bipustulatus*, *M. octodecimguttata*, *N. quadrimaculatus*, *N. cruentatus, R. cardinalis*, *S. syriacus*, *S. pallipediformis*, *S. gilvifrons* and *T. reduvinus*). Among the 19 species we found, 7 species were also reported from the native range of *M. hellenica* (Table A1 in Appendix). The number *of M. hellenica* predator species in all its distribution reached 40 after the present study. The updated predator list is provided in Table A1 (Appendix). On the other hand, our low sample size along with suboptimal assumption approach we adopted to identify predation on *M. hellenica* necessitate further studies focusing directly on the predatory behavior of the species found in this study in order to confirm their predatory status. We additionally provided eight new occurrence records for *M. hellenica* out of its native range in Turkey (Figure A1).

The western end of the Taurus Mountain chain is a significant geographical barrier separating the coastal Aegean and Mediterranean regions from the Burdur basin. Furthermore, the forest cover is not continuous into the inner regions which must be a major barrier for the spread of a weak disperser such as the giant pine scale (40 m/year) (Nicolopoulos 1965 cited in ref. [80]). Thus, human-aided introduction seems to be the only way for *M. hellenica* to spread the appropriate spots in the inner regions. The local beekeepers have been introducing *M. hellenica* to several areas in Burdur since 1990s but some of these efforts failed probably due to inappropriate ecological conditions for *M. hellenica*. However, the humid climate of the study site owing to the nearby lake seems to have facilitated its establishment. We found 12 new species predating upon *M. hellenica* in its introduced range and we could not find in the study site the other 12 species that had been reported as *M. hellenica* predators from its native range. The two ranges (native and introduced) had seven species in common (Table A1). Although some of the predators (particularly *N. kartliana*) might be accidentally introduced into the region during the introduction of *M. hellenica*, most of them were cosmopolitan species occurring also in *M. hellenica*-free ecosystems. Therefore, we believe that most of the predator species we identified have started foraging on *M. hellenica* in the past 30 years in the study site, although they most probably occur in the natural range of *M. hellenica*. These results also suggest that native predator community can rather quickly include the giant pine scale into the menu in scale’s introduced range. This is not surprising as prey switching is a strategy that predators use in the process of accepting a new prey species [17]. Although identifying the predation strategy of *M. hellenica* predators was out of the scope of the present study and its sampling design, we hypothesize that prey switching strategy might be used by most of the predators we identified. As a supporting observation for this hypothesis, the abundance of the Aphididae and Coccoidae species, which are the main preys of the predatory species we identified in the present study, was significantly low in the study region during all the field visits. Therefore, the most abundant prey species in the region which was always available to the predators was *M. hellenica*. This hypothesis should be tested by further studies.

The highest number of individuals we found belonged to *N. kartliana*. This result was in accordance with that of Ülgentürk et al. [19] who demonstrated that *N. kartliana* was the most common predator of *M. hellenica* in the entire range of the giant pine scale in Turkey. This is not surprising as *N. kartliana* is a monophagous predator [43]. On the other hand, it was found only in March, April and May 2019, but not in 2018 probably due to the sampling bias. Its pupae were available only in the cottony secretions of *M. hellenica* which were more abundant in 2019 because of trap-logs established for *O. erosus* management program. We found *N. kartliana* pupae in March and larvae by April. If the species were in the adult stage in late May (e.g., Gaimari et al. [54] found adults in June in Greece), it might escape from the sampling. Although revealing the biology of *N. kartliana* was out of our scope in this study, the abundance of it and its monophagous nature suggests that it is the best candidate for biological control against the giant pine scale as suggested by Avtzis et al. [14]. Considering observed high frequencies of *M. octodecimguttata* (68.4% of all field visits), *C. bipustulatus* (57.9%) and *H. quadripunctata* (47.4%), and absence or scarcity of other possible prey species in the study site, the hypothesis of these Coccinellids being significant suppressors of the giant pine scale populations seems reliable. However, this hypothesis remains for testing through future studies.

## 5 Conclusion

The giant pine scale is an important component of the biodiversity in its native range as it supports several insect species by its honeydew secretion. On the other hand, it can be a pest in habitats where the host trees are weak due to several reasons such as poor soil quality and climatic change. In such habitats, the presence of *M. hellenica* can favor outbreaks of secondary pests such as bark beetles which can eventually lead to death of the host trees [3,80]. Indeed, during the present study, we noted several *P. brutia* deaths due to the Mediterranean pine beetle particularly on the hosts heavily infested with the giant pine scale. Therefore, introducing *M. hellenica* out of its native range in favor of apiculture can lead to serious pest problems which may ultimately threaten not only the biodiversity but also the sustainability of pine honey production. Considering the serious impact of the climate change predicted for the eastern Mediterranean [81], control of *M. hellenica* can be expected to be one of the major issues in the near future in the pine forests of the region and in other introduction spots such as Australia. This makes research on *M. hellenica* predators and parasitoids, particularly those from Anatolia where the giant pine scale most probably originated from ref. [80,82], a priority. In the future studies related to biological control of populations of the giant pine scale, a special importance should be given to *N. kartliana* as it seems to be a host specific predator of the pest [14]. Additionally, since all the predatory species feeding upon *M. hellenica* described so far have been diurnal species, research on the nocturnal predatory species may also contribute to a list of giant pine scale predators.

## Appendix

**Table A1:** Predators of *Marchalina hellenica* identified so far in Georgia, Greece and Turkey (○: in the natural range of *M. hellenica* in TR, ●: in the introduced range of *M. hellenica* in TR)

Order: family	Species	Country	Reference
Acarina: Trombidiidae	*Allothrombium pulvinum* ○	Turkey	Ülgentürk et al. 2013 [19]
	*Allothrombium triticium* ○	Turkey	Ülgentürk et al. 2013 [19]
Acarina: Anystidae	*Anystis baccarum* ○●	Turkey	Ülgentürk et al. 2013 [19]; Present study
Neuroptera: Chrysopidae	*Chrysopa pallens* (Rambur)	Greece	Argyriou et al. 1976 [78]
	*Chrysopa (=Dichochrysa) flavifrons*	Greece	Argyriou et al. 1976 [78]; Nicolopoulos 1965 [80]
	*Chrysoperla carnea* ●	Turkey	Present study
	*Chrysoperla lucasina* ○	Turkey	Ülgentürk et al. 2013 [19]
	*Dichochrysa genei* ○	Turkey	Ülgentürk et al. 2013 [19]
	*Dichochrysa prasina* ○	Turkey	Ülgentürk et al. 2013 [19]
	*Pseudomallada flavifrons*	Greece	Nicolopoulos 1965 [80]
Neuroptera: Hemerobiidae	*Wesmaelius subnebulosus* ○	Turkey	Ülgentürk et al. 2013 [19]
Raphidioptera: Raphidiidae	*Raphidia ambigua* ●	Turkey	Present study
	*Raphidia* (=*Phaeostigma*) *notata*	Armenia, Georgia	Hadzibejli 1969 [89]
		Greece	Nicolopoulos 1965 [80]; Gaimari et al. 2007 [54]
Diptera: Chameamyiidae	*Leucopis* sp. ○	Turkey	Bodenheimer 1953; Selmi 1983 [24]
	*Leucopis obscura*	Greece	Nicolopoulos 1965 [80]
	*Neoleucopis kartliana* ○●	Turkey	Ülgentürk et al. 2013 [19]; Present study
		Georgia, Greece	Gaimari et al. 2007 [54]
Hemiptera: Anthocoridae	*Anthocoris nemoralis* ●	Turkey	Present study
	*Anthocoris nemorum* ●	Turkey	Present study
	*Brachysteles parvicornis* ○	Turkey	Selmi 1983 [24]
	*Cardiastethus nazarenus* ○	Turkey	Ülgentürk et al. 2013 [19]
	*Ectemnus* sp. ○	Turkey	Selmi 1983 [24]
	*Elatophilus pachycnemis* ○	Turkey	Ülgentürk et al. 2013 [19]
	*Orius majusculus* ●	Turkey	Present study
	*Temnostethus reduvinus* ●	Turkey	Present study
Hemiptera: Reduviidae	*Nagusta goedelii* ●	Turkey	Present study
Coleoptera: Coccinellidae	*Chilocorus bipustulatus* ○●	Turkey	Giray 1970 [23]; Selmi 1983 [24]; Present study
	*Coccinella septempunctata* ○	Turkey	Selmi 1983 [24]
	*Exochomus* sp.	Georgia	Hadzibejli 1969
	*Exochomus quadripustulatus* ○	Turkey	Selmi 1983 [24]
	*Harmonia quadripunctata* ○●	Turkey	Selmi 1983 [24]; Present study
	*Hippodamia variegata*	Greece	Nicolopoulos 1965 [80]
	*Myrrha octodecimguttata* ○●	Turkey	Ülgentürk et al. 2013 [19]; Present study
	*Nephus quadrimaculatus* ●	Turkey	Present study
	*Novius cruentatus* ●	Turkey	Present study
	*Rodolia cardinalis* ○●	Turkey	Ülgentürk et al. 2013 [19]; Present study
	*Scymnus pallipediformis* ●	Turkey	Present study
	*Scymnus subvillosus* ○●	Turkey	Ülgentürk et al. 2013 [19]; Present study
	*Scymnus syriacus* ●	Turkey	Present study
	*Stethorus gilvifrons* ●	Turkey	Present study
Coleoptera: Cantharidae	*Dasytes flavipes*	Turkey	Bodenheimer 1953 [22]
